# Clomiphene citrate stimulated cycles – additional gonadotrophin stimulation increases endometrium thickness without increasing implantation rate

**DOI:** 10.52054/FVVO.14.1.013

**Published:** 2022-04-03

**Authors:** I Magaton, A Helmer, M Roumet, P Stute, M Von Wolff

**Affiliations:** University Women’s Hospital, Division of Gynecological Endocrinology and Reproductive Medicine, Inselspital, Friedbühlstrasse 19, 3010 Bern, Switzerland; University of Bern, Clinical Trials Unit Bern, Mittelstrasse 43, 3012 Bern, Switzerland

**Keywords:** Endometrium, clomiphene citrate, minimal stimulation IVF, implantation rate, pregnancy rate, live birth rate

## Abstract

It is known that Clomiphene citrate (CC) reduces endometrial thickness, but it is unknown if additional gonadotrophin stimulation increases endometrial thickness and if this has an effect on implantation rate in in vitro fertilization (IVF).

The retrospective study included 263 minimal stimulation IVF-cycles stimulated with 25 mg CC per day (CC- IVF), and 161 IVF-cycles stimulated with CC plus 75IU hMG (human Menopausal Gonadotrophin) per day (CC/ hMG-IVF). Endometrial and oestradiol (E2) measurements were analysed between day -4 and 0 (0 = day of oocyte retrieval) and the association of endometrial thickness and treatment on implantation rates were studied after multiple adjustments.

It was shown that on day 0, endometrium was significantly thicker in CC/hMG-IVF versus CC-IVF cycles (9.81 ±2.68 versus 9.06 ±2.54 mm, p = 0.005). However, increased endometrial thickness did not have an effect on implantation and live birth rates.

In conclusion, gonadotrophins should not be added to low dose CC treated IVF cycles just to increase endometrial thickness as increased endometrial thickness does not increase implantation rate.

## Introduction

In the last decades, several steps have been made, aiming at rendering IVF-therapies more successful on one side, while also making them more supportable. Since its rise in popularity and global success, IVF still remains a complex and expensive therapy, especially considering its side effects and limited success rate. The development of minimal stimulation IVF, using clomiphene citrate (CC) and low dose gonadotrophins, is a step towards the objective of easier and less expensive IVF treatments.

CC is a frequently-used drug, not only in the case of anovulation, but also in minimal stimulation IVF (Teramoto and Kato, 2007; von Wolff et al., 2014; Abe et al., 2020). It reduces, even at dosages such as 25mg/day, effectively the risk of premature ovulations (von Wolff et al., 2014). It thereby increases transfer rates without the need of luteal phase support (von Wolff, 2019).

Despite its common use, several adverse effects of the CC treatment have been recognized. CC is known to suppress the endometrial estrogen and progesterone receptor concentration (Ronnberg et al., 1985). Ronnberg et al. also found that this effect is dose related: the antioestrogenic effect of CC on endometrium is less when 50 mg are administrated in comparison with 150 mg. Furthermore, CC decreases endometrial thickness despite a rise in serum estradiol (E2) (Imoedemhe et al., 1987). Since a thin endometrium (<8 mm) is recognized as a critical factor to achieve a pregnancy (Gonen et al., 1989; Gonen and Casper, 1990; Kasius et al., 2014), preventing CC-induced thinning of the endometrium can be assumed to be beneficial.

To reduce the adverse effect of CC on endometrial growth several approaches have been tested so far, such as adding hMG (human Menopausal Gonadotrophin) (Imoedemhe et al., 1987), ethinyl estradiol (Gerli et al., 2000) and estradiol valerate (Satirapod et al., 2013), demonstrating a reversed antiestrogenic effect on the endometrium. However, an implantation improving effect was either not analyzed or studied only in intrauterine insemination programmes. Furthermore, all studies involved CC at high doses of 100 to 150 mg daily.

Therefore, we performed a study to investigate whether in minimal stimulation IVF cycles with low dose CC stimulation the addition of low dose hMG increases endometrial thickness and if this has an effect on implantation and live birth rates.

## Methods

### Study population and participants

This is a retrospective observational single center study performed between 2015 and 2019. The study included 424 IVF cycles of women between 22-42 years of age with regular menstrual cycles (25- 35 days) and basal FSH concentration < 10 IU/L undergoing minimal stimulation IVF with daily dosages of 25mg CC per day (CC-IVF) or with CC in combination with 75IU hMG (CC/hMG-IVF). Women had been offered both kinds of therapy, but decided themselves which therapy they preferred to start with.

Women without embryo transfer, with endometriosis, with fibroids or other uterine pathologies and with sperm collection by testicular sperm extraction were excluded.

### IVF with CC and with CC plus 75IU hMG

CC-IVF patients received 25 mg CC per day, starting on day 4 of the cycle until an ovulation trigger was given. When the follicle diameter reached at least 18 mm and the E2 concentration was expected to be ≥ 1500 pmol/L, 5000 IU of human Chorionic Gonadotropin (hCG, Choriomon®, IBSA Institute Biochimique SA, Lugano, Switzerland) was administrated and patients were scheduled for an oocyte retrieval 36 hours later. The oocyte pick-up was performed using 19G single lumen needles. Follicles were flushed five times (Kohl Schwartz et al., 2020).

Fertilisation was performed by IVF or ICSI. Clinical pregnancy (number of amniotic sacs) and live birth rates were evaluated per transferred embryo.

CC/hMG-IVF patients were treated identically as CC-IVF patients but 75IU of hMG (Merional®, IBSA Institute Biochimique SA, Lugano, Switzerland) per day were added, starting on day 6 of the cycle until the day before an ovulation trigger was given.

All embryos were transferred 2 or 3 days after aspiration. All women performing CC/hMG-IVF received luteal phase support using 200mg vaginal micronized progesterone (Utrogestan ®, Vifor Pharma SA, Villars-sur-Glâne, Switzerland) per day. In CC-IVF luteal phase support was only given in case of a short luteal phase (<10 days).

692 endometrial thickness measurements were performed on day -4 to -0 (day -2 = day of ovulation trigger; day 0 = day of aspiration) in 263 CC-IVF and in 161CC/hMG-IVF cycles. E2 concentrations were analyzed on day -4 to -2.

### Statistical Analysis

For continuous cycle characteristic variables, mean and standard deviation, median and interquartile ranges, as well as minimum and maximum observed values were reported. Treatment groups were compared by chi-squared and non-parametric Wilcoxon tests for categorical and continuous variables respectively.

For endometrial thickness and oestrogen values mean and standard deviation, median and interquartile ranges, as well as minimum and maximum observed values are reported. Treatment groups were compared using non-parametric Wilcoxon tests.

The effects of different IVF treatments on the temporal evolution of the endometrial thickness were investigated separately using repeated measure models adjusted for age, cause of infertility, and number of previous embryo transfers without achieving a pregnancy. In these models, age, treatment, time and the interaction between time and treatment were included as fixed effects; patients and cycle within each patient were declared random effects.

The effect of endometrium thickness on clinical pregnancy and live birth per transferred embryo were analyzed using generalized estimating equation models for repeated measures, clustering in the patient level. To account for arbitrary correlation among observations within a patient we declared an exchangeable data correlation matrix and a robust variance estimator. Models were adjusted for the confounding effect of female age, type of treatment, cause of infertility, and number of previous embryo transfers.

The study was approved by the cantonal cantonal ethical committee Bern, Switzerland (KEK 2020- 00634), 26.05.2020.

## Results

Basic characteristics of the 226 women undergoing CC-IVF and/or CC/hMG-IVF treatment cycles are shown in Table I. Mean female age, infertility factors and number of previous embryo transfers without pregnancy were slightly different between the two groups.

**Table I:** Cycle characteristics stratified by treatment group (CC-IVF and CC/hMG-IVF).

	Total (n=424)	CC-IVF (n=263)	CC/hMG-IVF (n=161)	P-value
Female age at aspiration, years				0.005
Mean (SD)	35.6 (4.0)	35.1 (4.1)	36.3 (3.8)	
Median [IQR]	36.0 [33.0, 39.0]	36.0 [32.5, 38.0]	37.0 [33.0, 40.0]	
Range	23 - 42	23 - 42	25 - 42	
Causes of infertility, n (%)				<0.001
Male factor	170 (40.1%)	122 (46.4%)	48 (29.8%)	
Female factor	115 (27.1%)	66 (25.1%)	49 (30.4%)	
Male and female	82 (19.3%)	60 (22.8%)	22 (13.7%)	
Idiopathic	57 (13.4%)	15 (5.7%)	42 (26.1%)	
Number of previous embryo transfers without pregnancy, n (%)				<0.001
0-1	25 (5.9%)	6 (2.3%)	19 (11.8%)	
1-3	148 (34.9%)	87 (33.1%)	61 (37.9%)	
3-6	251 (59.2%)	170 (64.6%)	81 (50.3%)	
Number of retrieved oocytes				<0.001
Mean (SD)	1.71 (1.37)	1.37 (0.63)	2.27 (1.95)	
Median [IQR]	1 [1, 2]	1 [1, 2]	2 [1, 3]	
Range	0 - 13	0-5	0 - 13	
Number of transferred embryos				0.007
Mean (D)	0.94 (0.72)	0.87 (0.68)	1.08 (0.78)	
Median [IQR]	1 [0, 1]	1.00 [0, 1]	1 [1, 2]	
Range	0-3	0-3	0-3	
Clinical pregnancies, n (Pregnancy rate per transferred embryo, %)	51 (13.2%)	31 (13.7%)	20 (12.7%)	0.8
Live births, n (Born children per transferred embryo, %)	36 (9.5%)	23 (10.1%)	13 (8.6%)	0.6

Furthermore, Table I shows the number of retrieved oocytes, which was statistically significant different between CC-IVF and CC/ hMG-IVF, and the number of embryos transferred.

**Figure 1:**
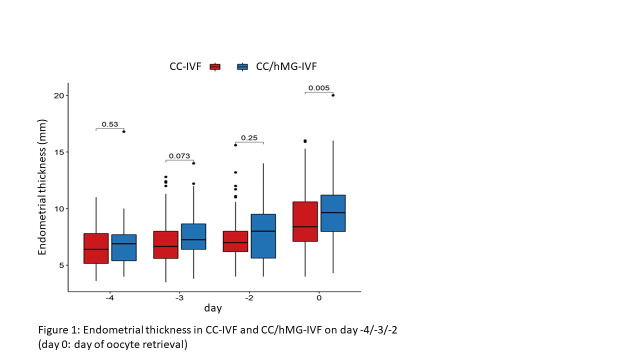
Endometrial thickness in CC-IVF and CC/hMG-IVF on day -4/-3/-2 (day 0: day of oocyte retrieval).

Endometrial thickness increased in CC- IVF from 6.65 ±1.70mm on day -4 to 9.06 ±2.54mm on day 0 and in CC/hMG-IVF from 7.00 ±2.32mm to 9.81 ±2.68mm, respectively. Endometrial thicknesses between the two groups was significantly different on day 0, the day of oocyte retrieval (p = 0.005) but not on day -4, -3 and -2 (Table II, Figure 1).

**Table II:** Treatment characteristics on day -4/-3/-2/0 (day 0 = day of oocyte aspiration).

	Day -4				Day -3				Day -2				Day 0			
	Total, (n=110)	CC-IVF (n=76)	CC/hMG-IVF (n=34)	P-value	Total, (n=139)	CC-IVF (n=77)	CC/hMG-IVF (n=62)	P-value	Total, (n=125)	CC-IVF (n=82)	CC/hMG-IVF (n=43)	p-value	Total, (n=424)	CC-IVF (n=263)	CC/hMG-IVF (n=161)	p-value
Endometrium (mm)				0.5				0.073				0.3				0.005
Mean (SD)	6.76 (1.91)	6.65 (1.70)	7.00 (2.32)		7.37 (2.20)	7.15 (2.20)	7.63 (2.19)		7.64 (2.21)	7.54 (2.19)	7.83 (2.26)		9.33 (2.61)	9.06 (2.54)	9.81 (2.68)	
Median [IQR]	6.50 [5.38,7.80]	6.40 [5.15,7.80]	6.90 [5.40,7.70]		7.00 [5.82,8.28]	6.65 [5.60,8.00]	7.25 [6.40,8.65]		7.30 [6.00,8.70]	7.00 [6.20,8.00]	8.00 [5.62,9.50]		9.00 [7.50,11.00]	8.40 [7.10,10.60]	9.65 [7.97,11.20]	
E2 (pmol/L)				<0.001				<0.001				<0.001				
Mean (SD)	1’391 (1’007)	979 (466)	2’275 (1’265)		2’012 (1’258)	1’448 (738)	2’708 (1’415)		2’071 (1’155)	1’705 (643)	2’777 (1’545)					
Median [IQR]	1’054 [764, 1’669]	850 [680, 1’187]	1’959 [1’308, 2’892]		1’666 [1’087, 2’462]	1’224 [950, 1’684]	2’440 [1’713, 3’452]		1’831 [1’330, 2’418]	1’591 [1’230, 2’020]	2’380 [1’788, 3’023]					

E2 concentration were significantly higher in CC/ hMG stimulated cycles on all days analysed.

Clinical pregnancy and live birth rates per transferred embryo were not different in CC-IVF and CC/hMG-IVF cycles. Clinical pregnancy rates were 13.7% versus 12.7% (p = 0.8), and live birth rates 10.1% versus 8.6% (p = 0.6), respectively (Table I).

Endometrium thickness did not have an effect on clinical pregnancy rate (aOR 1.04, 95% CI 0.92, 1.18, p = 0.52) and live birth rate (aOR 1.05, 95% CI 0.9, 1.21; p = 0.55).The odds ratios were adjusted for female age, cause of infertility and number of previous transfers.

## Discussion

Our study showed that the addition of low dose hMG to CC stimulated cycles can reverse the thinning effect of low dose CC on endometrium. Our study also revealed that additional hMG stimulation does not increase implantation rate, indicating that the gonadotropin induced artificial increase of endometrial thickness does not seem to have substantial effects on endometrial function. In minimal stimulation IVF CC is used to reduce the risk of premature ovulations and to stimulate folliculogenesis to increase the number of retrieved oocytes. Dosages of 25 mg CC (CC25) or 50 mg (CC50) per day are applied, starting at the beginning of the follicular phase and continued until the day of HCG trigger (Teramoto and Kato, 2007; von Wolff et al., 2014; Abe et al., 2020).

The strength of the study is that the effects of CC and hMG has been analyzed in IVF treatments without embryo selection and with fresh embryo transfers and not in intrauterine insemination programmes as in previous studies, which allowed a precise calculation and comparison of implantation rates. Furthermore, our study focused on low dose CC stimulation, applied until the day of hCG trigger, this protocol has not been studied before. Accordingly, our study evaluated a stimulation regime which is similar to the ones used in minimal stimulation IVF (Teramoto and Kato, 2007; von Wolff et al., 2014; Abe et al., 2020).

The cumulative clomiphene dose used in our study can be assumed to be similar to common protocols with 50 mg CC for 5 days.

A major weakness of our study is its retrospective design requiring multiple adjustments to reduce the risk of a bias.

The endometrium thinning effect of CC seem to depend on the CC dosage. In CC25-IVF cycles, CC reduces endometrial thickness by 0.5 mm compared to spontaneous cycles (7.9 ±1.6mm vs 8.4 ±1.7mm) (von Wolff et al., 2014). In CC50 cycles with CC given for 5 days, endometrial thickness is reduced in the late proliferative phase by 1.3mm compared to spontaneous cycles (9.5 ±1.7mm vs 10.8 ±2.6mm, p=0.025) and in the mid secretory phase by 0.3mm (10.0 ±1.8mm vs 10.3 ±2.9mm) (Dehbashi et al., 2003).

Concerning current literature, our results are in line with other studies, also showing that increased E2 concentrations can reverse the endometrium thinning effect of CC. In fact, to reduce the adverse effect of CC on endometrial growth several approaches have been tested so far, such as adding hMG (Imoedemhe et al., 1987), ethinyl estradiol (Gerli et al., 2000) or estradiol valerate (Satirapod et al., 2013). However, in contrast to our study, previous studies did not evaluate pregnancy rates following IVF treatments. Furthermore, most studies applied CC at much higher doses such as 100 to 150 mg daily.

## Conclusion

The addition of 75IU of hMG in CC25 stimulated minimal stimulation IVF cycles, applied until the day of hCG trigger, reverses the endometrium thinning effect of CC but without increasing the implantation rate. This indicates that gonadotropin induced artificial increased endometrial thickness does not seem to improve the implantation potential of the endometrium. Therefore, gonadotropins can be added to increase the number of retrieved oocytes but should not be added exclusively to increase endometrial thickness to increase implantation rates.
